# Early Detection of Incipient Retinal Pigment Epithelium Atrophy Overlying Drusen with Fundus Autofluorescence *vs*. Spectral Domain Optical Coherence Tomography

**DOI:** 10.1155/2020/9457457

**Published:** 2020-09-16

**Authors:** Anabel Rodríguez, Marc Biarnés, Rosa M. Coco-Martin, Anna Sala-Puigdollers, Jordi Monés

**Affiliations:** ^1^Institut de la Màcula Centro Médico Teknon, Barcelona, Spain; ^2^Barcelona Macula Foundation, Barcelona, Spain; ^3^Instituto de Oftalmobiología Aplicada (IOBA), Universidad de Valladolid, Valladolid, Spain; ^4^Red Temática de Investigación Cooperativa en Salud de Oftalmologia (Oftared), Instituto de Salud Carlos III, Madrid, Spain; ^5^Institut Clínic d'Oftalmologia (ICOF), Hospital Clínic, Barcelona, Spain

## Abstract

**Purpose:**

This study aims to find out which tool, fundus autofluorescence (FAF) or spectral domain optical coherence tomography (SD-OCT), is more sensitive in detecting retinal pigment epithelium (RPE) demise overlying drusen and can, therefore, help predict geographic atrophy (GA) appearance in Age-Related Macular Degeneration (AMD).

**Methods:**

A single-site, retrospective, observational, longitudinal study was conducted. Patients with intermediate AMD (iAMD) (large (>125 *μ*m) or intermediate (63–125 *μ*m) drusen with hyper/hypopigmentation) with a minimum follow-up of 18 months were included. Drusen with overlying incipient RPE atrophy were identified on SD-OCT defined as choroidal hypertransmission or nascent geographic atrophy (nGA). These selected drusen were, then, traced backwards in time to determine if incipient RPE atrophy overlying drusen was observed on FAF (well-demarcated region of absence of autofluorescence) before, simultaneously, or after having detected the first signs of incipient RPE atrophy on SD-OCT. The number of drusen in which signs of incipient RPE atrophy was detected earlier using FAF or SD-OCT was compared. The time elapsed from the identification with the more sensitive method to the other was recorded and analyzed.

**Results:**

One hundred and thirty-three drusen in 22 eyes of 22 patients were included. Of these, 112 (84.2%) drusen showed choroidal hypertransmission and 21(15.8%) nGA. Early signs of atrophy overlying drusen were found simultaneously on SD-OCT and FAF in 52 cases (39.1%, 95% CI 30.8–47.9%), earliest on FAF in 51 (38.3%, 95% CI 30.0–47.2%) and first on SD-OCT in 30 (22.6%, 95% CI 15.8–30.6%; *p* < 0.05). Statistically significant differences were found between both techniques (*p*=0.005), with FAF detecting it earlier than SD-OCT. When RPE atrophy was found first on FAF, the median time to diagnosis with SD-OCT was 6.6 months (95% CI 5.5 to 8.6), while if detection occurred earlier on SD-OCT, the median time until identification with FAF was 12.6 months (95% CI 6.0 to 23.4; *p*=0.0003).

**Conclusions:**

In iAMD cases in which early atrophy overlying drusen is not detected simultaneously in FAF and SD-OCT, FAF was significantly more sensitive. Nevertheless, a multimodal approach is recommended and required to evaluate these patients.

## 1. Introduction

The hallmark of the intermediate stage of age-related macular degeneration (AMD) is the presence of drusen [1]. Drusen are deposits of extracellular material located between the basement membrane of the retinal pigment epithelium (RPE) and the inner collagenous layer of Bruch's membrane [2]. They can cause mild metamorphopsia and decreased sensitivity in microperimetry or dark adaptation, among other clinical symptoms. As drusen increase in number or size, the disease progresses and the risk of vision loss increases. In the late stages of AMD, the disease advances towards neovascular AMD and/or geographic atrophy (GA) usually inducing great vision worsening [3, 4].

In the last two decades, new imaging techniques have been incorporated to study AMD, and one of such techniques is fundus autofluorescence imaging (FAF), which provides information about RPE integrity in a noninvasive way [5]. Different studies have shown that, in early or intermediate stages of the disease, the FAF image has the capacity to show RPE alterations in normal-appearing fundus regions [5–8]. On FAF, GA appears as a well-demarcated region of marked hypoautofluorescence due to the absence of the fluorophore lipofuscin contained within the RPE [7–11]. GA usually appears in the central or parafoveal macula and spreads centrifugally [8, 12]. New areas of atrophy may show a relatively lower intensity of hypoautofluorescence. FAF imaging is especially valuable in GA because it better delineates the areas of discrete or small GA in comparison with other imaging modalities [11, 13].

On the other hand, spectral domain optical coherence tomography (SD-OCT) facilitates *in vivo* high-resolution evaluation of the retina [14]. GA has been extensively studied with SD-OCT, and alterations within the atrophic area and its borders have been described in detail, hypertransmission of OCT signal below Bruch's membrane being one of the best recognized findings [14–18]. Besides, Guymer et al. used SD-OCT to describe precursors of GA. They defined nascent geographic atrophy (nGA) by the presence of either the subsidence of the outer plexiform layer (OPL) and the inner nuclear layer (INL) and/or a wedge-shaped band within the boundaries of the OPL [18–20].

Additionally, Wu et al. investigated FAF in areas of nGA and areas of drusen-associated atrophy and concluded that areas of nGA can present as both hyper- and hypoautofluorescent changes, while in drusen-associated atrophy, most often appeared as hypoautofluorescent areas [19].

Currently, GA has no treatment, but detecting its earliest signs can help understand its natural course and assist in the characterization of the disease spectrum. This would also improve the design of clinical trials to develop preventive or therapeutic strategies. Therefore, knowing which one of two commonly used imaging methods for monitoring GA, FAF, and SD-OCT is more sensitive for detecting the earliest signs of the disease is relevant for future advances in this field.

In the present study, we aim to compare two imaging techniques, SD-OCT and FAF, to determine which one is more sensitive to detect incipient RPE atrophy overlying drusen in patients with iAMD.

## 2. Materials and Methods

### 2.1. Design and Participants

This is a retrospective, observational, longitudinal study conducted at the Institut de la Màcula (Hospital Quirón Teknon; Barcelona, Spain). The study adhered to the tenets of the Declaration of Helsinki and was approved by the Fundación Quirón Salud Ethics Committee. All patients signed informed consent.

Charts of patients with a diagnosis of iAMD visited between January 2010 and October 2014 were reviewed, and the last date of follow-up was July 2017. The drusen type of interest was soft drusen.

All patients met the following inclusion criteria: male or female patients, over 50 years of age, diagnosed with iAMD (AREDS stage 2 or 3: large (>125 *μ*m) or intermediate (63–125 *μ*m) drusen and associated hyper-/hypopigmentation), with a minimum follow-up of 18 months after diagnosis. Patients were excluded if the studied eye included any prior history of neovascular AMD, >0.5 disc areas of RPE atrophy (1.27 mm^2^), other concomitant macular diseases (macular edema and retinal dystrophies), spherical equivalent greater than ± 6′00 D, previous history of intraocular treatment (laser photocoagulation and intravitreal injections) or surgery (with the exception of phacoemulsification), concomitant use of medications known to be toxic to the retina (chloroquine, hydroxychloroquine, and tamoxifen), or SD-OCT image quality < 20. The presence of reticular drusen in the studied eye was not considered a reason for exclusion.

### 2.2. Procedures

All patients received a complete ophthalmic examination by an experienced retina specialist. The checkup included medical history, best-corrected visual acuity (BCVA) measured in logMAR, and intraocular pressure measured with Goldmann applanation tonometry and indirect fundus ophthalmoscopy. Imaging exam entailed FAF (excitation 488 nm, absorption >500 nm) and SD-OCT imaging (Heidelberg Spectralis^TM^ HRA + OCT, Heidelberg Engineering, Heidelberg, Germany), alongside fovea-centered nonstereoscopic 30° color fundus retinography (TRC-50DX, Topcon Medical Systems, Tokyo, Japan). The SD-OCT exam was performed using a high-resolution volumetric scanning protocol centered on the fovea (19 or 37 horizontal B-scans). Alongside, a high-resolution (1536 × 1536 pixels) infrared FAF image covering a 30° angle of the fundus and centered on the fovea was recorded.

**Figure 1:**
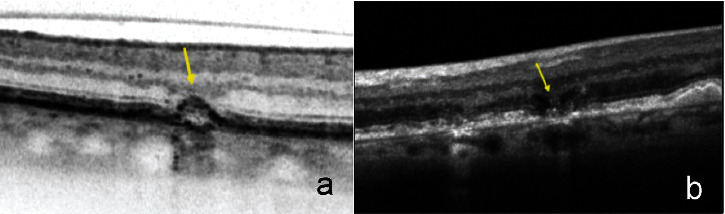
SD-OCT features. (a) B-scan of SD-OCT where drusen with hypertransmission are observed; (b) B-scan of SD-OCT showing nascent geographic atrophy.

Upon review of the Heidelberg Spectralis database of the Institut de la Màcula, an experienced observer (AR) selected and classified the patients with iAMD who met the aforementioned eligibility criteria. Then, individual soft drusen showing early signs of RPE atrophy overlying them were identified. This was defined by the presence of any of the following:

1. Hypertransmission: drusen that show increased hypertransmission signal below Bruch's membrane on SD-OCT, which indicates a loss of overlying RPE (Figure 1(a))
2. Nascent GA (nGA): subsidence of the outer plexiform layer (OPL)/inner nuclear layer (INL) and/or a hyporeflective wedge-shaped band within the boundaries of the OPL (Figure 1(b))

**Figure 2:**
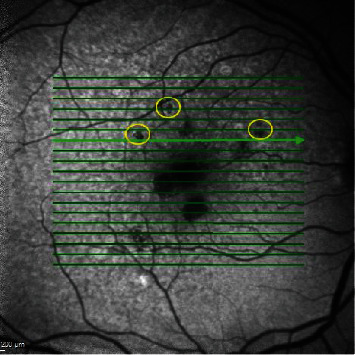
An example of retinal pigment epithelium atrophy by fundus autofluorescence. The yellow circle shows a region of absence of autofluorescence.

RPE atrophy on FAF was defined as a well-demarcated region of absence of autofluorescence (Figure 2).

The color fundus retinography was used to verify that the absence of autofluorescence was not caused by pigment clumping over drusen. Drusenoid pigment epithelium detachments (defined in the AREDS studies as a well-defined, pale yellow or white, large mound consisting of many large drusen or confluent drusen with ≥350 *µ*m in the narrowest diameter) [21] were excluded.

When a drusen presented hypertransmission or nGA, previous and subsequent SD-OCT and FAF images were reviewed. The first data in which the absence of autofluorescence on FAF was observed was, then, recorded. There were three possible scenarios:

- The first time that the absence of autofluorescence on FAF was observed coincided with the date in which atrophy of the RPE was first detected by SD-OCT. Thus, the diagnosis of incipient RPE atrophy overlying drusen was simultaneous with both imaging methods.
- The absence of autofluorescence on FAF was present before RPE atrophy was detected by SD-OCT. Thus, FAF identified incipient RPE atrophy overlying drusen earlier than SD-OCT.
- The absence of autofluorescence on FAF was appreciated after RPE atrophy detection by SD-OCT. Thus, SD-OCT identified incipient RPE atrophy overlying drusen earlier than FAF.
- When in doubt, a second experienced observer (AS) reevaluated the case and the decision of the second evaluator was taken as the outcome for the analysis.

### 2.3. Main Outcome Measures

The primary endpoint was the comparison of the number of drusen in which there was incipient RPE atrophy overlying drusen detected earlier by FAF than by SD-OCT *vs*. the number of drusen in which this occurred earlier on SD-OCT than on FAF. The number of drusen in which RPE atrophy was detected simultaneously on both imaging modalities was also recorded.

The secondary endpoint included the time elapsed from the identification with the more sensitive method to identification with the other in cases of nonsimultaneous identification of early atrophy overlying drusen.

### 2.4. Statistical Analysis

Univariate statistics were used to describe the sample using mean and standard deviation (SD) for quantitative and number and percentage for categorical variables. The unit of analysis was each soft drusen contained in the 20° × 30°, fovea-centered SD-OCT macular grid. The percentage of time in which RPE atrophy overlying drusen was detected earlier by SD-OCT or by FAF was determined and compared using Fisher's exact test.

### 2.5. Secondary Endpoints

When one imaging method detected incipient RPE atrophy overlying drusen earlier than the other, the Kaplan–Meyer plots were used to estimate the median time to detection with the second, less-sensitive tool. The time when one method detected RPE atrophy for the first time (either FAF or SD-OCT) was considered time 0. The logrank test was used to compare both curves.

In cases where FAF detected RPE atrophy earlier than SD-OCT, the time to detection of RPE atrophy with SD-OCT, using either the definition of hypertransmission or nGA, was determined. The results were again compared using the logrank test.

The intra- and interobserver agreement for FAF detection of RPE atrophy were determined using the kappa index (ĸ) in a randomly selected sample of 35 drusen. The ĸ measures agreement between categorical observations was adjusted for chance.

Data analysis was conducted using Stata IC, version 15.1 (StataCorp, Texas, USA). A two-tailed *p* value <0.05 was considered statistically significant.

## 3. Results

One hundred and fifty-one drusen from 22 eyes in 22 patients with iAMD showed hypertransmission or nGA after a minimum follow-up of ≥18 months and were initially enrolled. Eighteen of these drusen (seven patients) were excluded: eight could not be assessed by being present in a region with dense macular pigment and ten by incomplete clinical information. Therefore, 133 drusen from 22 patients were finally included in the analysis. The mean age of these patients was 71.1 ± 6.9 years, 93.3% being female and all being Caucasian. The number of drusen included per eye ranged from 1 to 27, with a mean of 13.9 ± 8.8. The mean baseline BCVA of the studied eyes was 0.11 ± 0.13 logMAR (equivalent to approximately 20/25 in Snellen notation). Eighteen out of the 22 eyes (81.8%) were phakic at baseline, and the rest were pseudophakic. Only one phakic eye (1/18, 5,6%) became pseudophakic during the follow-up. Given the small number of pseudophakic eyes, the results were largely driven by the phakic sample and no stratified analysis could be conducted.

**Figure 3:**
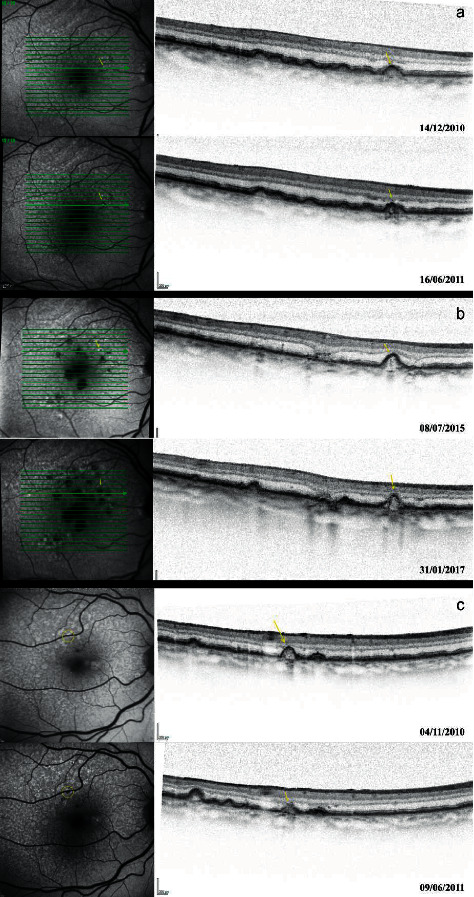
Examples of simultaneous detection, earlier detection with FAF, and earlier detection with SD-OCT. FAF: fundus autofluorescence; RPE: retinal pigment epithelium; and SD-OCT: spectral domain optical coherence tomography. (a) Simultaneous detection (the incipient RPE atrophy was observed at the same time with both exams). In the top image (14/Dec/2010), the selected druse (yellow arrow) showed normal autofluorescence on FAF and no hypertransmission on SD-OCT. In the next visit (16/Jun/2011), incipient atrophy of the RPE by both imaging techniques was observed. (b) Earlier detection with FAF (RPE atrophy overlying drusen is detected earlier on FAF than on SD-OCT). The top image (08/Jul/2015) shows that while a marked area of absence of autofluorescence appears on FAF, no signs of RPE atrophy were detected with SD-OCT. In the bottom image (31/Jan/2017), after 18 months, signs of atrophy on SD-OCT can be observed and the area of atrophy on FAF increased in size. (c) Earlier detection with SD-OCT. In the top image (04/Nov/2010) there is SD-OCT hypertransmission (yellow arrow), but normal FAF. Seven months later (09/Jun/2011), the absence of autofluorescence was noticeable (bottom image).

Incipient RPE atrophy overlying drusen was observed simultaneously on both tests in 52/133 drusen (39.1%, 95% CI 30.8% to 47.9%), while early RPE loss was detected first by FAF in 51/133 (38.40%, 30.1% to 47.2%) and first by SD-OCT in 30/133 (22.6%, 95% CI 15.8% to 30.6%), as seen in Table 1 and Figure 3. Statistically significant differences between early detection with FAF or using SD-OCT were found (*p*=0.005), being FAF more sensitive.

**Figure 4:**
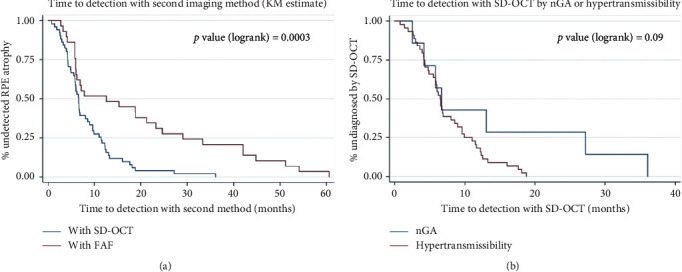
Comparison of time to secondary detection of incipient GA. FAF: fundus autofluorescence; nGA: nascent geographic atrophy; SD-OCT: spectral domain optical coherence tomography. (a) On the left-hand side, the Kaplan–Meyer curves of time to detection with SD-OCT when FAF detected atrophy earlier (blue) and with FAF when SD-OCT detected atrophy earlier (red) are shown. (b) On the right-hand side, the Kaplan–Meyer estimates compare if earlier detection was made by showing choroidal hypertransmission or nGA when SD-OCT detected the atrophy.

Considering only cases in which atrophy detection was not simultaneous in both imaging modalities, the median time from initial detection with FAF to subsequent detection with SD-OCT was 6.6 months (95% CI, 5.5 to 8.6 months); and it was 12.6 months (95% CI, 6.0 to 23.3 months) from detection with SD-OCT to later detection with FAF.  Figure 4(a) shows these differences being statistically significant (*p*-value = 0.0003).

Taking into account only the detection of incipient atrophy using SD-OCT, we found choroidal hypertransmission in 112/133 of the cases (84.5%, 95% CI 76.9% to 90.0%) and nGA in 21/133 (15.5%, 95% CI 10.0% to 23.1%). Therefore, the odds ratio (OR) of identifying incipient RPE atrophy overlying drusen by choroidal hypertransmission in comparison with nGA was 5.33 (95% CI 3.16 to 9.01, *p* value <0.0001).

Considering only cases in which atrophy detection was not simultaneous in both imaging modalities, detection through choroidal hypertransmission was made with a median of 6.5 months after the detection of incipient atrophy with FAF (95% CI, 4.9 to 8.6 months) and detection through nGA after a median of 6.7 months (95% CI, 2.5 to 27.3 months; *p* value = 0.09), differences not being statistically significant (Figure 4(b)).

Finally, the consistency within and between observers in grading the presence or absence of incident RPE atrophy as measured with FAF was determined by the kappa index. The intraobserver agreement, determined for just one of the evaluators (AR), was 100%, with kappa = 1.00 (95% CI, 1.00 to 1.00; *p* value < 0.0001). The interobserver agreement was 91.4%, with kappa = 0.62 (95% CI, 0.23 to 1.00; *p* value < 0.0001).

## 4. Discussion

This study compared FAF and SD-OCT in the detection of incipient RPE atrophy overlying drusen in patients with iAMD. We chose to compare FAF with SD-OCT because they are the standard diagnostic tests used in the detection, evaluation, and monitoring of GA. Atrophic AMD does not have treatment, so understanding the evolution at the early stages of the disease with current imaging techniques may be key to future advances.

The results of this study show that detection of early atrophy overlying drusen was observed simultaneously on both imaging techniques about 40% of the time (Figure 3(a)). Therefore, in 60% of the occasions, one method detected signs of incipient atrophy earlier than the other (Figures 3(b) and 3(c)). This suggests that a multimodal approach including both FAF and SD-OCT would be recommendable for early detection of atrophy overlying drusen.

When atrophy was detected just on one imaging method, FAF detected it earlier than SD-OCT (38.4% vs 22.6%, *p*=0.005). True differences favoring FAF may be related to the advantages of using an *en face* modality to visualize changes in the retina as opposed to the cross-sectional nature of structural SD-OCT, in which protocols with wide interscan distances may miss the point of early atrophy if the B-scan is not located precisely in the location of the RPE loss. Given that even very dense protocols have a distance between adjacent B-scans in the range of tenths of microns, combined use of FAF with SD-OCT increases the likelihood of detection of early signs of GA. We also excluded the drusen located closer to the foveola due to the impossibility to differentiate the absence of autofluorescence caused by macular pigment absorption from that caused by true early atrophy, and this fact may have favored an increased sensitivity of detection using FAF.

Also, once atrophy was detected only with one imaging modality, time to detection with the other method differed markedly (*p*=0.0003). It took approximately 6 months to detect incipient atrophy with SD-OCT after FAF detected it, while detection with FAF once it was detected with SD-OCT took approximately 12 months. These differences are not easily explained taking into account that, in almost half of the sample, the detection of atrophy was simultaneous with both imaging modalities. Certainly, differences between the follow-up times of each patient hinder the estimation of the precise moment in which atrophy appears on each of the individual drusen. On the other hand, we can speculate that the underlying mechanism leading to RPE loss may differ between distinct drusen, making some imaging modalities to be more readily apt to detect incipient atrophy than other. In fact, using fluorescence lifetime imaging ophthalmoscopy (FLIO) eyes with drusen showed longer autofluorescence lifetimes than healthy controls, and different lifetime values were found in different drusen, suggesting a heterogeneous ultrastructural composition in phenotypically similar lesions [22].

In the vast majority of cases in which atrophy was detected with SD-OCT, choroidal hypertransmission was observed more frequently than nGA (84% vs. 16%, *p* < 0.0001), although the median time to detection with either phenomenon was not significantly different. This suggests that hypertransmission may be a precursor of nGA. This could be expected since hypertransmission arises as an immediate consequence of tissue loss, whereas nGA is detected after subsidence of inner retinal tissue and the appearance of the hypereflective wedge-shaped band, which arise as a consequence (not as a primary cause) of a certain amount of tissue loss. Besides, like other authors [23], here, we demonstrate that nGA is a predictor for GA providing supportive evidence of the potential value of nGA as a surrogate endpoint in future intervention trials for the early stages of AMD.

Everyone agrees that multimodal imaging is the gold standard nowadays to study AMD. Other new techniques that should be evaluated and compared in the future with those tested here would include the optical coherence tomography angiography (OCT-A) and the *en face* OCT. OCT-A allows for three-dimensional visualization of retinal blood flow and, in recent studies, has shown choriocapillaris flow alterations particularly associated with the development of GA that exceed atrophy boundaries spatially and that are a prognostic factor for future GA progression. Besides, OCT-A may be helpful to differentiate GA from mimicking diseases [24]. Furthermore, *en face* OCT imaging could also be useful for identifying areas suspicious for nGA, in differential diagnosis, study design, and patient assessment [25, 26].

However, this study has limitations that need to be acknowledged. First, this is a retrospective study, although the fact that patients are studied backwards to the time when the retinal images were normal provides some prospective nature to the study. Second, the number of eyes was small, but our unit of analysis was the numerous individual drusen observed. Third, drusen within the foveal area were excluded to avoid interference caused by the presence of the luteal pigment in the evaluation of the FAF, so the detection of incipient atrophy in this region may be easier with SD-OCT because its signal is not interfered as much by the luteal pigment as it is with FAF. Also, detection with SD-OCT could have been improved by the use of more dense volume protocols (decreased distance between B-scans that may have had an increased chance of crossing a focal area of RPE loss), but anyway, this is always a limitation with a cross-sectional device. It remains to be determined if the use of *en face* strategies at different heights could have improved the SD-OCT detection rate. Pseudofaquia influence could not be checked because of the small number of pseudophakic eyes. Finally, the patients were visited at irregular intervals, and therefore, estimates of time to appearance of RPE atrophy may be overestimated.

In any case, FAF imaging allows greater accuracy of border identification, revealing patterns predictive of growth rates once the GA is stated in advanced AMD [27], but here, we demonstrate that it is also the best tool to detect it early. Our study suggests that FAF is more sensitive that SD-OCT as a biomarker that could help to predict individual disease progression from early to advanced AMD. These finding could be useful to plan artificial intelligence diagnostic tools and to test new treatments for atrophic AMD.

## 5. Conclusions

In summary, 40% of the drusen in the iAMD showed signs of incipient RPE cell loss over them simultaneously in the FAF and SD-OCT. Incipient GA was initially detected only in one of the two imaging methods in 60%. In the latter case, the FAF detected signs of atrophy earlier than SD-OCT showing the absence of autofluorescence. While SD-OCT can detect signs of atrophy indistinctly through the observation of both, choroidal hypertransmission or nGA, we should learn to recognize these patterns. Therefore, multimodal imaging in the iAMD including both examination tools is recommended to detect signs of incipient GA as soon as possible.

## Figures and Tables

**Table 1:** Percentage of drusen showing incipient atrophy of the retinal pigment epithelium detected first with each imaging tool.

Imaging exam detecting first GA signs	*n*	Percentage^*∗*^ (95% CI)
Fundus autofluorescence	51/133	38.4 (30.1 to 47.2)
SD-OCT	30/133	22.6 (15.8 to 30.6)
*Simultaneously on both exams*	52/133	39.1 (30.8 to 47.9)

^*∗*^Percentages do not add up to 100% due to rounding. CI: confidence interval; SD-OCT: spectral domain optical coherence tomography.

## Data Availability

The data used to support the findings of this study are available from the corresponding author upon reasonable request.
